# Efficacy of frovatriptan versus other triptans in the acute treatment of menstrual migraine: pooled analysis of three double-blind, randomized, crossover, multicenter studies

**DOI:** 10.1007/s10072-012-1044-7

**Published:** 2012-05-30

**Authors:** Gianni Allais, Vincenzo Tullo, Stefano Omboni, Chiara Benedetto, Grazia Sances, Dario Zava, Michel D. Ferrari, Gennaro Bussone

**Affiliations:** 1 Department of Gynecology and Obstetrics, Women’s Headache Center, University of Turin, Via Ventimiglia 3, 10126 Turin, Italy; 2 Department of Clinical Neurosciences, Carlo Besta National Neurological Institute, Milan, Italy; 3 Italian Institute of Telemedicine, Varese, Italy; 4 Headache Centre, IRCCS C. Mondino Foundation, Institute of Neurology, Pavia, Italy; 5 Istituto Lusofarmaco d’Italia, Milan, Italy; 6 Department of Neurology, Leiden Centre for Translational Neuroscience, Leiden University Medical Centre, Leiden, The Netherlands

**Keywords:** Almotriptan, Menstrually related migraine, Frovatriptan, Rizatriptan, Zolmitriptan

## Abstract

The objective of this study was to review the efficacy and safety of frovatriptan (F) versus rizatriptan (R), zolmitriptan (Z) and almotriptan (A), in women with menstrually related migraine (IHS criteria) through a pooled analysis of three individual studies. Subjects with a history of migraine with or without aura were randomized to F 2.5 mg or R 10 mg (study 1), F or Z 2.5 mg (study 2), and F or A 12.5 mg (study 3). The studies had an identical multicenter, randomized, double-blind, crossover design. After treating three episodes of migraine in no more than 3 months with the first treatment, patients had to switch to the next treatment for other 3 months. 346 subjects formed intention-to-treat population of the main study; 280 of them were of a female gender, 256 had regular menses and 187 were included in the menstrual migraine subgroup analysis. Rate of pain free at 2, 4 and 24 h was 23, 52 and 67 % with F and 30, 61 and 66 % with comparators (*P* = NS). Pain relief episodes at 2, 4 and 24 h were 37, 60 and 66 % for F and 43, 55 and 61 % for comparators (*P* = NS). Rate of recurrence was significantly (*P* < 0.05) lower under F either at 24 h (11 vs. 24 % comparators) or at 48 h (15 vs. 26 % comparators). Number of menstrual migraine attacks associated with drug-related adverse events was equally low (*P* = NS) between F (5 %) and comparators (4 %).

## Introduction

In more than 50 % of women with migraine, the migraine attack is often associated with the menstrual cycle [1, 2]. These headache attacks are reported to be particularly severe, more disabling, more difficult to manage, and need immediate acute or preventive treatment with a drug capable of ensuring a sustained effect [3].

The efficacy and safety of triptans in the management of menstrual migraine, either as acute therapy or intermittent prophylaxis, have been demonstrated in numerous randomized clinical trials [4]. Following this evidence, these drugs are now recommended as first-line treatment for menstrual migraine [5, 6].

Frovatriptan is an antimigraine agent of the triptan class developed in order to provide a triptan with the clinical potential for a long duration of action and a low likelihood of side effects and drug interactions [7]. Recently, post hoc analyses of three double-blind, randomized, crossover, head-to-head trials have compared the efficacy and safety of frovatriptan with that of rizatriptan [8], zolmitriptan [9] and almotriptan [10] in women with menstrual migraine. These studies showed a similar efficacy of frovatriptan, rizatriptan, zolmitriptan and almotriptan in the immediate treatment of menstrual migraine, but lower recurrence rates, and thus a better sustained relief under frovatriptan.

In the present paper, we report on results of a pooled efficacy and safety analysis of frovatriptan versus the comparators in menstruating women based on the aforementioned publications.

## Methods

### Study population and design

The original study design of the three studies, including patient’s selection criteria, is detailed in the original publications [8–10]. Briefly, the studies recruited subjects of both genders, aged 18–65 years, with a current history of migraine with or without aura, according to the International Headache Society definition [11], and with at least one, but no more than 6 migraine attacks per month for 6 months prior to entering the study. The analysis of this subgroup population was predefined in the statistical analysis plan and original protocols of the three studies. This condition was defined according to the IHS research criteria, as migraine without aura attacks in a menstruating woman, occurring on day 1 ± 2 (namely days −2 to +3) of menses in at least two out of three menstrual cycles and additionally at other times of the cycle [11].

The studies had a multicenter, randomized, double-blind, crossover design and involved 33 different centers across Italy. Each patient received frovatriptan 2.5 mg or rizatriptan 10 mg in the first study [8], frovatriptan 2.5 mg or zolmitriptan 2.5 mg in the second study [9] and frovatriptan 2.5 mg or almotriptan 12.5 mg in the third study [10] in a randomized sequence. After treating a maximum of three episodes of migraine in no more than 3 months with the first treatment, the patient switched to the other treatment and was asked to treat a maximum of three episodes of migraine in no more than 3 months with the second treatment.

The study involved three visits and each patient’s participation time in the study was not to exceed 6 months from randomization. Subjects having no migraine episodes during one of the two observation periods were excluded from the study.

Randomization was done by blocks of four. Blindness was ensured by the overencapsulation technique, i.e., by inserting study drug tablets in capsules.

### Data analysis

This pooled analysis was carried out in all menstruating women randomized to any of the two treatment sequences foreseen in each study, enrolled to receive either study treatment and having treated at least one episode of menstrual migraine with both medications in each study.

The following endpoints were evaluated [11]: (a) the proportion of pain relief episodes at 2, 4 and 24 h (a decrease in migraine intensity from severe or moderate to mild or none at 2, 4 and 24 h); (b) the proportion of pain free episodes at 2, 4 and 24 h (the absence of migraine episodes at 2, 4 and 24 h after intake of one dose of study drug); (c) recurrence within 24 h (episodes pain free at 2 h and headache of any severity returns within 24 h); (d) recurrence within 48 h.

Safety analysis was applied to the intention-to-treat population, by calculating the incidence of drug-related adverse events.

Continuous variables were summarized by computing average values and standard deviations (SD), while categorical variables by computing the absolute value and the frequency (as percentage). Study endpoints were compared between groups by a *t* test of Student (continuous variables) or by a Chi-square test (categorical variables). Kaplan–Meier curves for the cumulative hazard of recurrence over the 48 h were also drawn. The level of statistical significance was kept at 0.05 for all analyses.

## Results

### Baseline demographic and clinical data

The main study population consisted of 346 subjects, of whom 280 were women and 236 in the fertile age [8–10]. A total of 187 out of the 236 eligible women treated at least one episode of menstrual migraine with both medications and were thus included in the present analysis.

Demographic and clinical baseline data of the 346 patients of the three main studies pooled together and of the subgroup of 187 women with menstrually related migraine are reported in Table 1. No statistically significant differences were observed between the whole study population and the subgroup.

**Table 1:** Demographic and clinical baseline data of the 346 patients of the three main studies pooled together and of the subgroup of 187 women with menstrually related migraine

	Main studies (*n* = 346)	Subgroup of menstruating women (*n* = 187)	*P*
Age (years, mean ± SD)	38 ± 10	36 ± 8	NS
Height (cm, mean ± SD)	166 ± 7	164 ± 6	NS
Weight (kg, mean ± SD)	64 ± 13	61 ± 9	NS
Age at onset of migraine (years, mean ± SD)	17 ± 7	16 ± 6	NS
Migraine attack duration >2 days (*n*, %)	72 (21)	42 (22)	NS
No use of triptans in the previous 3 months (*n*, %)	146 (42)	83 (44)	NS
Moderate or severe attacks (*n*, %)^a^	1,574 (80)	327 (82)	NS
Patients with at least one moderate or severe attack (*n*, %)	334 (97)	179 (96)	NS

Data are shown as mean (±SD) or absolute (*n*) and relative frequency (%)

^a^Numbers refer to number and frequency of attacks with respect to overall number of attacks

### Efficacy results

A total of 401 out of the overall 1,978 attacks were classified as menstrually related: 199 (20 %) were treated with frovatriptan and 202 (20 %) with comparators (66 women treated with rizatriptan, 54 with zolmitriptan and 67 with almotriptan).

As summarized in Table 2, at 2, 4 and 24 h the rates of pain relief episodes were not significantly (*P* = NS) different between frovatriptan (37, 60 and 66 %) and the comparators (43, 55 and 61 %, respectively). Also, the proportions of pain free episodes at 2, 4 and 24 h did not differ (*P* = NS) between treatments (23, 52 and 67 % frovatriptan vs. 30, 61 and 66 % comparators).

**Table 2:** Main study endpoints in the two study treatment groups (frovatriptan and other triptans)

	Frovatriptan	Comparators	*P*
Pain relief episodes at 2 h	74 (37)	87 (43)	NS
Pain free episodes at 2 h	46 (23)	60 (30)	NS
Pain relief episodes at 4 h	120 (60)	113 (55)	NS
Pain free episodes at 4 h	104 (52)	124 (61)	NS
Pain relief episodes at 24 h	133 (66)	124 (61)	NS
Pain free episodes at 24 h	133 (67)	133 (66)	NS
Recurrent episodes at 24 h	22 (11)	49 (24)	<0.05
Recurrent episodes at 48 h	29 (15)	53 (26)	<0.05

Data are reported as absolute (*n*) and relative (%) frequency. *P* refers to the statistical significance of the difference between the two study drugs

**Fig. 1:**
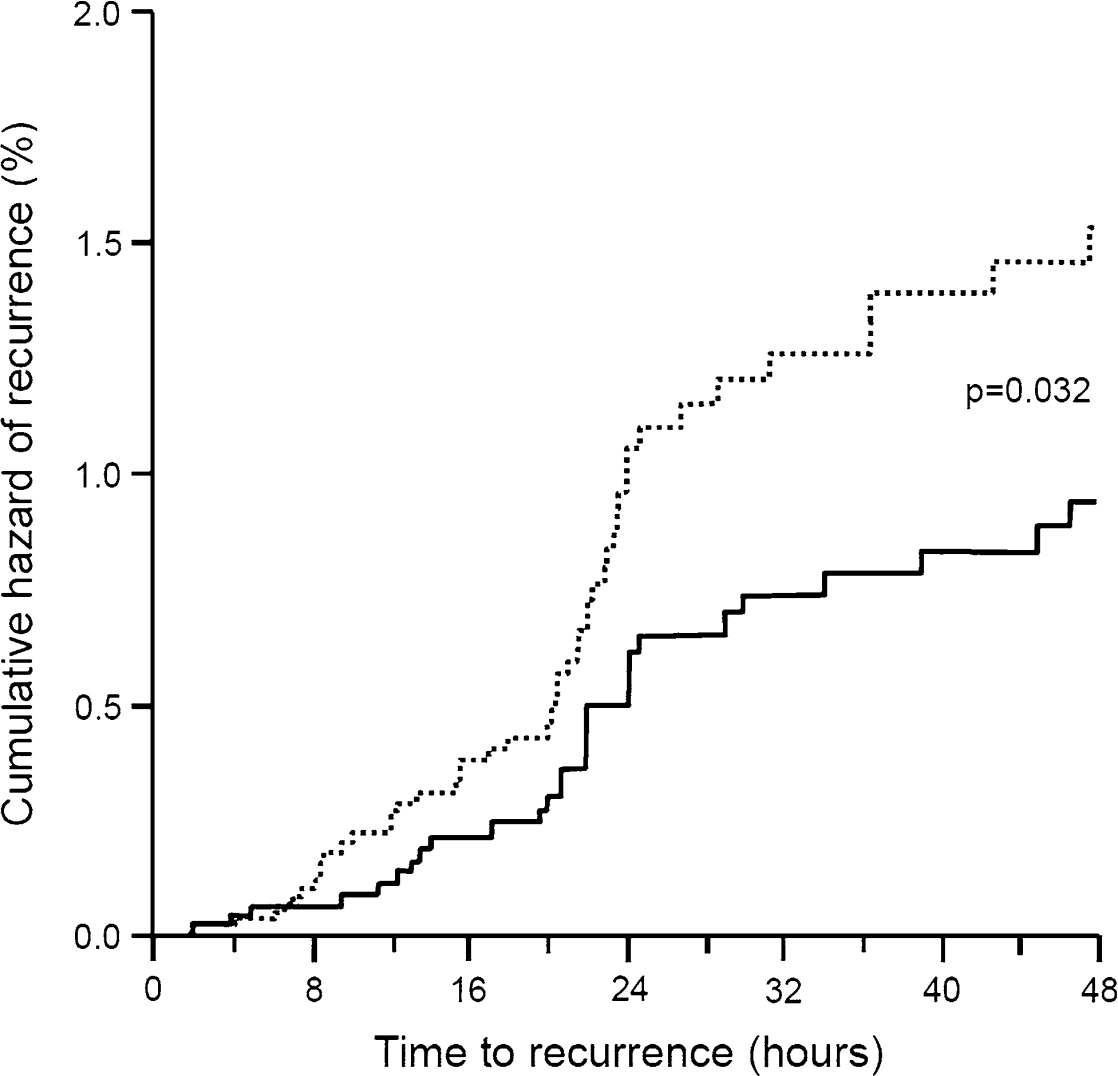
Cumulative hazard of recurrence over 48 h during treatment with frovatriptan or comparators, in the 187 patients of the intention-to-treat (ITT) population. Data are shown separately for frovatriptan (*continuous line*) and for the three comparators pooled together (*dotted line*). *P* value refers to the statistical significance of the between-treatment difference

Conversely, the rate of recurrent episodes at 24 h was significantly (*P* < 0.05) lower under frovatriptan (11 vs. 24 % with comparators, Table 2). This was also the case for recurrence at 48 h (15 % frovatriptan vs. 26 % comparators, *P* < 0.05, Table 2). Differences in cumulative hazard of recurrences over the 48 h were in favor of frovatriptan (Fig. 1).

### Safety results

A total of 18 drug-related adverse events were recorded in 401 treated menstrually related attacks. No statistically significant differences were observed in the rate of attacks associated with drug-related adverse events between frovatriptan (10/189 attacks, 5 %) and the comparators (8/194, 4 %).

## Discussion

In this pooled analysis of three double-blind, randomized, direct comparative, crossover studies [8–10], acute treatment of menstrually related migraine with frovatriptan and other triptans (rizatriptan, zolmitriptan and almotriptan), resulted in similar proportions of pain relief and pain free episodes at 2, 4 and 24 h. Despite a similar immediate antimigraine efficacy profile of the studied drugs, frovatriptan showed a more sustained relieving effect on migraine, with lower headache recurrence rates over 24 h and even more so over 48 h. Such differences might be explained, at least in part, by differences in the pharmacokinetics of frovatriptan with respect to the other triptans. Frovatriptan has a longer elimination half-life than rizatriptan, zolmitriptan and almotriptan, this possibly explaining why frovatriptan, unlike the other tested triptans, greatly reduced the risk of recurrence [12].

This is the first analysis of head-to-head, double-blind, randomized trials of frovatriptan versus other triptans in women suffering from menstrual migraine. Our study and a retrospective analysis of almotriptan versus zolmitriptan are the only available double-blind, randomized studies comparing the efficacy of two triptans [13]. In a previous publication treatment of 136 women with almotriptan 12.5 mg and of 119 women with zolmitriptan 2.5 mg resulted in similar proportions of 2 h pain relief and pain free as well as 2–24 h recurrences between the two groups [13].

Though we acknowledge that the strength of our results might be weakened by the post hoc nature of the analysis, no such prospective studies are yet available or have been planned. Our results encourage the design and implementation of larger direct comparative randomized clinical trials evaluating triptan efficacy in female migraineurs.

In terms of safety, in our pooled analysis, treatment with frovatriptan and other triptans was associated with a similar low prevalence of adverse drug reactions. This reinforces evidence from prior placebo controlled or head-to-head trials, namely that frovatriptan, used for immediate or repeated sustained use, is one of the best tolerated among triptans [14–19].

In conclusion, our analysis of individual data of double-blind, randomized, crossover trials suggests that frovatriptan and other widely employed triptans share a similar efficacy in the immediate treatment of acute attack of menstrual migraine. However, frovatriptan seems to offer the advantage of a lower risk of recurrence and thus a more sustained effect than the other triptans.

## Appendix

Coordinators: G. Bussone (Milano), B. Fierro (Palermo), L. Pinessi (Torino).

Investigators: P. De Martino (Torino), B. Panascia (Palermo), R. Rapisarda (Palermo), F. Devetag (Feltre), M.G. Sances (Pavia), L.A. Pini (Modena), G. Bono (Varese), R. Cerbo (Roma), M. De Marinis (Roma), M. Guidotti, R. Ravasio (Como), M. Alessandri (Grosseto), E. De Caro (Catanzaro), F. Lanaia (Catania), M.P. Prudenzano (Bari), M. Gionco (Torino), A. Aguggia (Novi Ligure), B. Colombo (Milano), M. Turla (Esine), F. Perini (Vicenza), A. Ganga (Sassari), E. Agostoni (Lecco), C. Narbone (Messina), A. Moschiano (Merate), M. Vacca (Cagliari), M. Bartolini (Ancona), A. Ambrosini (Pozzilli), R. De Simone (Napoli), V. Petretta, F. D’Onofrio (Avellino), M. Bartolini (Ancona), M.A. Giamberardino (Chieti), C. Lisotto (San Vito al Tagliamento), P. Martelletti (Roma), D. Moscato (Roma), L. Savi (Torino), P. Santoro (Monza), G. Zanchin (Padova), B. Fierro (Palermo), D. Pezzola (Istituto Lusofarmaco d’Italia, Milano), G. Reggiardo (Biostatistical Unit, Mediservice, Milano), F. Sacchi (Clinical Unit, Mediservice, Milano).
